# WSSNet: Aortic Wall Shear Stress Estimation Using Deep Learning on 4D Flow MRI

**DOI:** 10.3389/fcvm.2021.769927

**Published:** 2022-01-24

**Authors:** Edward Ferdian, David J. Dubowitz, Charlene A. Mauger, Alan Wang, Alistair A. Young

**Affiliations:** ^1^Department of Anatomy and Medical Imaging, University of Auckland, Auckland, New Zealand; ^2^Auckland Bioengineering Institute, University of Auckland, Auckland, New Zealand; ^3^Department of Biomedical Engineering, King's College London, London, United Kingdom

**Keywords:** 4D Flow MRI, computational fluid dynamics, deep learning, wall shear stress (WSS), aorta

## Abstract

Wall shear stress (WSS) is an important contributor to vessel wall remodeling and atherosclerosis. However, image-based WSS estimation from 4D Flow MRI underestimates true WSS values, and the accuracy is dependent on spatial resolution, which is limited in 4D Flow MRI. To address this, we present a deep learning algorithm (WSSNet) to estimate WSS trained on aortic computational fluid dynamics (CFD) simulations. The 3D CFD velocity and coordinate point clouds were resampled into a 2D template of 48 × 93 points at two inward distances (randomly varied from 0.3 to 2.0 mm) from the vessel surface (“velocity sheets”). The algorithm was trained on 37 patient-specific geometries and velocity sheets. Results from 6 validation and test cases showed high accuracy against CFD WSS (mean absolute error 0.55 ± 0.60 Pa, relative error 4.34 ± 4.14%, 0.92 ± 0.05 Pearson correlation) and noisy synthetic 4D Flow MRI at 2.4 mm resolution (mean absolute error 0.99 ± 0.91 Pa, relative error 7.13 ± 6.27%, and 0.79 ± 0.10 Pearson correlation). Furthermore, the method was applied on *in vivo* 4D Flow MRI cases, effectively estimating WSS from standard clinical images. Compared with the existing parabolic fitting method, WSSNet estimates showed 2–3 × higher values, closer to CFD, and a Pearson correlation of 0.68 ± 0.12. This approach, considering both geometric and velocity information from the image, is capable of estimating spatiotemporal WSS with varying image resolution, and is more accurate than existing methods while still preserving the correct WSS pattern distribution.

## Introduction

Wall shear stress (WSS) is an important contributor to vessel wall remodeling and atherosclerosis (1–3). WSS is defined as the shear force produced by tangential blood flow on the vessel wall as a result of blood viscosity and is related to the gradient of velocity in the surface normal direction. Previous studies suggest that wall shear stress is an important biomarker for atherosclerosis formation (4, 5). Both low and high time-averaged WSS (TAWSS) have been suggested to be associated with pathology. Moreover, recent studies also found that a high oscillatory shear index (OSI) plays an important role in causing wall thickening (6). Early detection of these biomarkers may provide useful information for clinical practice. However, despite recent findings on the importance of WSS and related measures, there is yet no practical method to accurately measure WSS from clinical data.

Magnetic resonance imaging (MRI) phase contrast imaging methods enable non-invasive quantification of the three-dimensional time varying velocity field (4D Flow MRI) (7, 8). However, the spatiotemporal resolution of 4D Flow MRI is limited. Several existing methods have employed curve fitting to estimate WSS from velocity derivatives near the vessel wall (9–11). Stalder et al. (9) introduced a velocity-based method using B-spline interpolation. A later study investigated several approaches based on velocity mapping, Fourier velocity encoding, and intravoxel velocity SD mapping (12). Overall, all these methods are dependent on spatial resolution, segmentation accuracy, velocity encoding (VENC), and voxel position relative to the wall, with each method being more sensitive to different parameters. These methods show consistent reproducibility when the comparison between methods was performed relative to each other. However, the WSS obtained using these methods were consistently lower compared to the values obtained from computational fluid dynamics (CFD) (13, 14). This underestimation is likely due to the limited resolution of 4D Flow MRI, as hemodynamic parameters may be biased due to partial volume effects and temporal blurring. The need to have a higher resolution MRI is constrained by the limited amount of examination time.

Computational modeling enables physics-based simulation of clinical data at high resolution, constrained only by computation resources. While it is possible to achieve accurate estimates for hemodynamic variables, CFD simulations require patient-specific parameters. These boundary conditions are not always obtainable and often rely on assumptions such as vessel rigidity, incompressible fluid, and pressure estimations. Moreover, the amount of computation required to solve the numerical problem is often not feasible in a clinical setting.

Recent developments in medical imaging and deep learning have enabled the use of physics-based simulations as surrogates to train a deep learning model (15–18). These approaches offer high accuracy compared to the CFD ground truth by learning spatial representation of geometric features. Liang et al. (18) used a shape decoding technique to train a network to estimate aortic stress distributions based on the input mesh. Similarly, Acebes et al. (16) presented a CNN-based network to estimate endothelial cell activation potential (ECAP) using an unwrapped model of the left atria. Gharleghi et al. (15) also presented a deep learning method to estimate TAWSS in left main coronary bifurcations by using geometric information as the input. Conversely, conventional curve fitting methods have used velocity information at constant spatial locations (equidistant inward normal). By combining the use of CFD simulations, variable geometric features, velocity information, and deep learning, a fast and accurate method that can be applied to *in vivo* data can be developed.

This study proposes a deep learning approach to estimate WSS based on patient-specific aortic vessel geometries and velocity information. To achieve this, CFD simulations were generated for patient-specific geometries in order to extract a uniform grid sampling of spatial and velocity information at two inward distances from the aortic vessel wall. The locations of the sampled velocity sheets were encoded as coordinate flatmaps and varied over a range of values, enabling the network to learn the relationships between geometry, sampling distance, velocity, and WSS. WSS vectors were output as a uniform-grid flatmap, predicted at any given time frame, enabling the calculation of other WSS measures, such as TAWSS and OSI. The method was validated on synthetic 4D Flow MRI data derived from the CFD simulations. Additionally, the method was applied to clinical *in vivo* cases in comparison with the parabolic fitting method.

## Methods

The following section provides a detailed description of the methodology used in this study. First, the data generation process is described, including the geometry extraction from 4D Flow MRI, CFD simulation setup, and input data preparation step. Second, the description of WSSNet is presented, including the network architecture, loss function definition, and training hyperparameters setup. Finally, the performance of the network is evaluated with respect to the estimated WSS magnitudes, WSS distribution, time-averaged WSS (TAWSS), and OSI, with quantifications performed in the CFD dataset, synthetic MRI from CFD, and actual *in vivo* cases.

### Geometry Extraction

Clinical cardiac 4D Flow MRI were obtained for a total of 59 volunteers and patients using a prototype sequence. Data were acquired using a 1.5T scanner (MAGNETOM Avanto fit, Siemens Healthcare, Erlangen, Germany). 4D Flow images were acquired with retrospective gating, encoding velocities of 150 cm/s (VENC) at 2.375 mm grid spacing, 2.75 mm slice thickness, covering the entire heart and great vessels. Other parameters included repetition and echo time (TR/TE) of 38.3 and 2.3 ms, respectively, and flip angle 7^o^, with 38–58 ms temporal resolution and ~20 reconstructed frames.

Sixteen cases were excluded due to low image quality, leaving 43 cases for this study (34 healthy, 9 left ventricular hypertrophy). Patient-specific aortic geometries were extracted from the 43 *in vivo* cases. Phase contrast magnetic resonance angiography (PC-MRA) images (temporal mean) were constructed to define the anatomical structure. For each case, two segmentations were performed, one with aortic branches, and another one without. We refer to the segmentation without branches as the “*aorta-only-segmentation*.” For consistency, the branch segmentations contained 3 aortic branches: brachiocephalic artery (BCA), left common carotid artery (LCCA), and left subclavian artery (LSA). For the aorta-only-segmentation, these branches were simply excluded. Segmentations were performed semi-automatically using the ITK-SNAP (19) active contour method. The resulting segmentations were exported as a surface mesh.

The 3D aortic segmentations (ones with the aortic branches) were truncated at the ascending aorta distal to the aortic root and at the distal end of the thoracic aorta to obtain flat inlet and outlet surfaces to be used for CFD simulations. The aortic branches were also truncated ~2 cm from their bases. An additional smoothing operation (10–12 iterations of vertices' distance averaging) was applied to smooth out the rough surface obtained from the segmentation. All these steps were performed using Blender 2.8 (20). Finally, Instant Meshes (21) was utilized to retopologize the complex meshes to more structured quad surfaces. This set of geometries was used to perform the CFD simulations.

**Figure 1:**
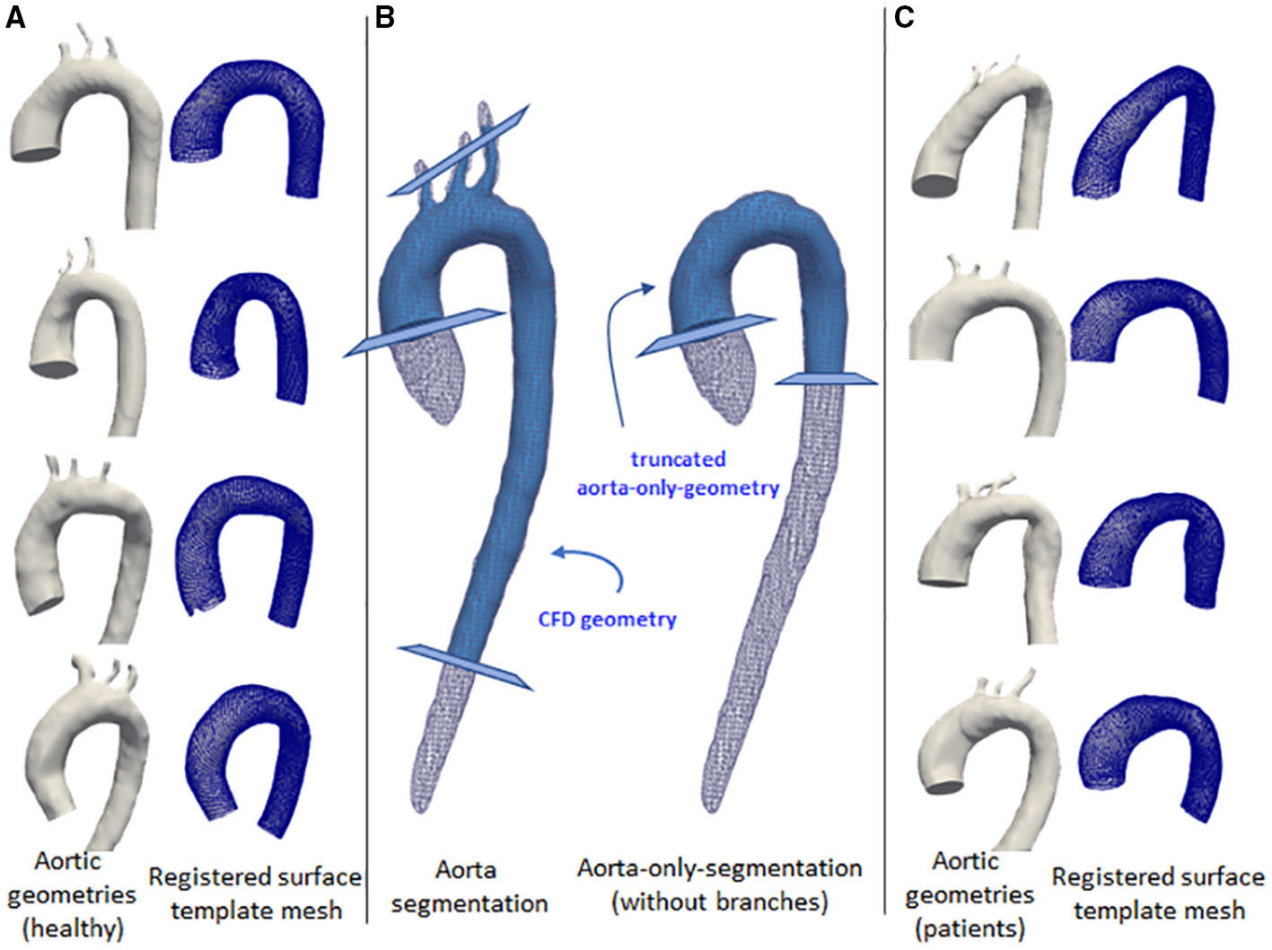
Some examples of the aortic geometry in the dataset. The first and third columns show the corresponding aorta (left) and the registered surface template mesh (right). The first column **(A)** shows aorta geometries for normal volunteers. Third column **(C)** show aorta geometries from left ventricular hypertrophy cases. The middle column **(B)** shows the aortic segmentation with branches (left) and aorta-only-segmentation (right), shown with white wireframes. Light blue geometries show the processed segmentation for the use of computational fluid dynamics (CFD) simulation and mesh registration. The cross-sectional planes show the truncation lines for the geometry marking the inlet and outlets.

The same steps were also applied for the aorta-only-segmentations (ones without the branches), except that they were truncated at around the mid-thoracic level of the descending aorta. This second set of geometries was used for the registration step using a surface template mesh, as explained below. Figure 1 shows the two geometries and the locations of the truncation-lines.

### CFD Simulations

The branched aortic geometries were imported to Ansys 19.2 (Canonsburg, PA, USA). A mesh independence study was performed on one of the geometries from the training set (case 12), over four grid resolutions (1.5, 1.0, 0.75-, and 0.5-mm tetrahedral elements) using a steady-state simulation. For all the four grid resolutions, the same boundary layer setup was used (1 mm total thickness, 10 prism layers with increasing thickness, the growth rate of 1.2). Inlet and outlets were refined with 0.3 mm tetrahedral elements. The selected meshing strategy (1 mm elements, 589k nodes, 1.5M elements) compared to the finer mesh (0.75 mm elements, 746k nodes, 1.9M elements) resulted in differences of <4% for average WSS, <3% for average velocity, and <2% for flow rate at three cross-sectional planes measured at the ascending aorta, aortic arch, and descending aorta. Accordingly, this meshing strategy was selected considering the computation cost, file export size, and computational resource availability.

All 43 geometries were discretized using the selected meshing strategy, with additional local refinements applied when necessary. This results in a mesh containing between 500k and 800k nodes for each geometry.

We imposed rigid and no-slip boundary conditions at the wall. Blood was modeled as a Newtonian fluid with a density of 1,060 kg/m^3^ and a viscosity of 4 × 10^−3^ Pa.s. A plug flow profile was prescribed at the inlet. Two different variations of outlet boundary conditions were prescribed: (1) constant pressure (0 Pa) was set at all outlets, (2) flow percentage ratio, with 70% flow going to the descending aorta, and 15/5/10% going to BCA, LCCA, and LSA, respectively. Due to time and resource constraints, the different outlet boundary conditions (1) and (2) were applied separately for 25 and 18 geometries, respectively.

Time-varying patient-specific inflow velocity was extracted from one case (case 1) over a cardiac cycle (710 ms). All simulations were performed for two cardiac cycles, using the same time-periodic velocity profile. The simulations were run with a time step of 1 ms. Velocity and wall shear stress vectors were extracted from the last cardiac cycle of the simulation to avoid transient initialization effects. The data were obtained for every 10th time step (dt = 10 ms), resulting in 72 time frames (71 from the last cycle and 1 from the last time frame of the previous cycle).

It is important to note in this study that while patient-specific velocity profiles could be acquired, the rationale of running these CFD simulations was to generate a dataset with sufficient flow variations to enable the network to learn the local relationships between velocity and WSS, mainly through the different geometries and temporal variation. Hence, the same boundary conditions were applied for all geometries. Consequently, the resulting CFD simulations were not compared against the actual measurements from 4D Flow MRI. As a result, the WSS obtained from CFD simulations were different from the *in vivo* cases, which was expected. Therefore, the network applicability in predicting unseen data can be tested.

Essentially, WSS can be formulated as


$$\begin{array}{l} \tau_{w}=\mu \frac{\partial u}{\partial y} \end{array}$$

which is a product of the dynamic viscosity of the fluid (*u*) and the velocity gradient near the wall (wall shear rate). While in general, blood flow in the aorta is laminar and during peak systole, the flow can become turbulent, specifically at the ascending aorta (22). In turbulent flow, the velocity gradients near the wall become very steep and, hence, also the wall shear stress, as the velocity follows a logarithmic profile. The use of the laminar model for aortic flow is known to underestimate WSS (23). For this reason, the use of a turbulence model helps to improve WSS calculations through the use of turbulence (eddy) viscosity, which is a part of turbulence computations.

The realizable k-ε turbulence model (24) was chosen to account for possible turbulence effects during the peak systolic phase, where the Reynolds number reached >5,000. The incompressible Navier-Stokes equations were solved iteratively in ANSYS Fluent 19.2, with convergence criteria of scaled residual value to be less than 10^−5^ for mass and momentum. Each simulation took between 40 and 50 core hours to solve on a high-performance parallel computing environment (1.5 GB/core).

### Data Preparation

Due to the complex relationship between flow and velocity gradients, it is important to incorporate both the velocity and spatial information as inputs to the network. Node coordinates, velocity, and WSS vectors from CFD were processed to create pairs of input-output data for the network.

**Figure 2:**
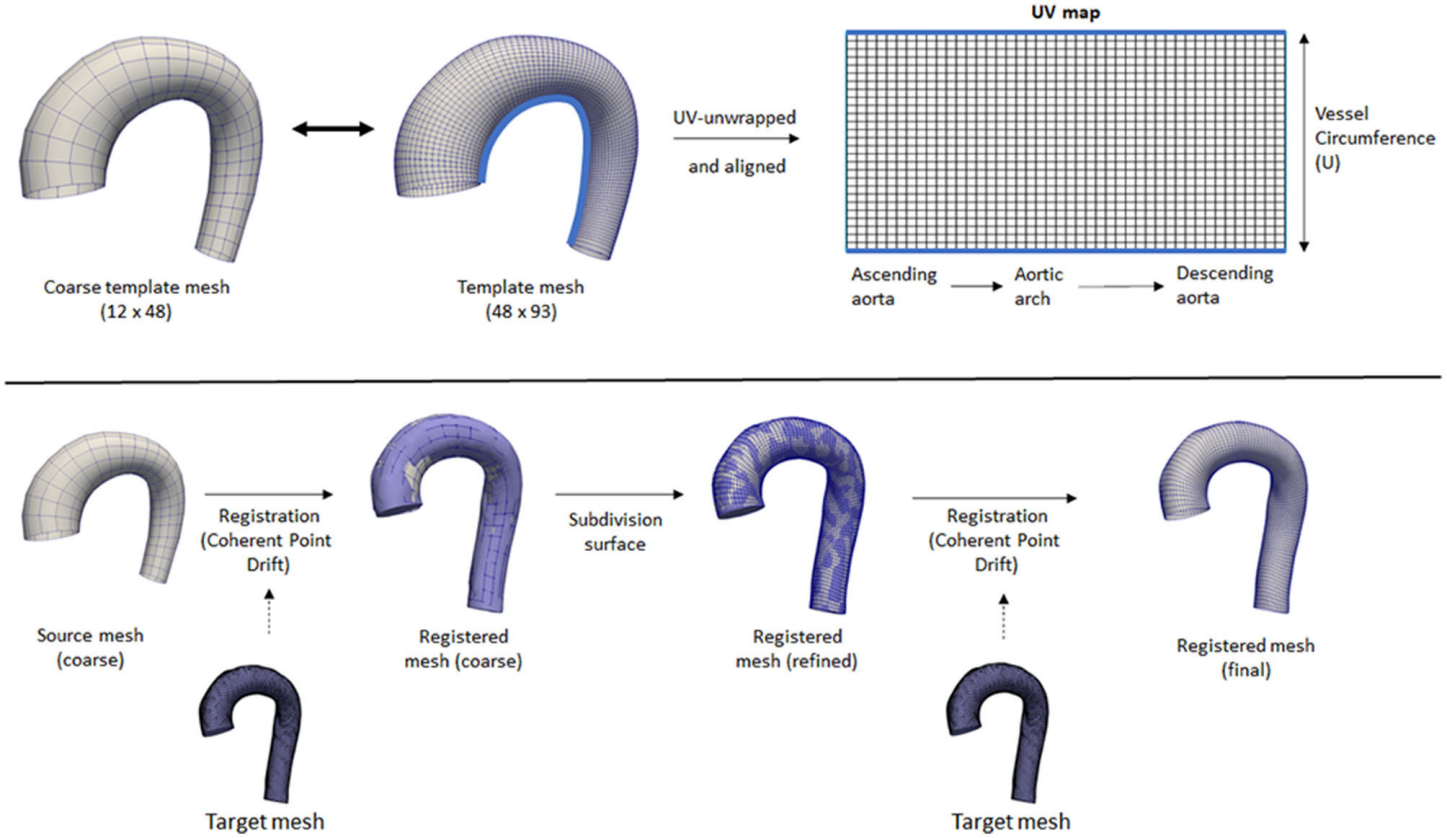
Top: Coarse and fine template meshes used for registration. UV unwrapping was performed on the fine template mesh, with a light blue line showing the cut line. Bottom: An overview of the registration process using Coherent Point Drift. Registration was performed on the coarse template mesh, followed by a subdivision surface operation, followed by another registration step on the refined mesh.

To have a standardized data structure, we utilized the surface template mesh representation from Liang et al. (18, 25), which was modified into a 48 × 93 quadrilateral mesh. The quadrilateral mesh was then unwrapped into a UV map, and a 2D flatmap representation with 48 and 93 corresponding to the size of the circumference (U) and longitudinal (V) directions, respectively. The template mesh extends from the ascending aorta, aortic arch, and proximal section of the descending aorta. Note that the template mesh did not model the branching vessels. The template mesh was unwrapped using Blender, with the shortest distance from ascending to descending aorta selected as the cut-line. Subsequently, the UV map was aligned to form rectangular elements. To speed up the mesh registration step, a coarse version of the template (12 × 24) was also constructed and paired with subdivision matrices to convert it back to its actual size (48 × 93) using subdivision surface (26). These two template meshes were used for registration. Figure 1 shows the variations of geometry used to build the training dataset and each geometry is shown in pairs: the aortic geometry with branches used for the CFD simulations, and the registered mesh on the aorta-only-geometry used for the WSSNet. Comprehensive visualization of the template mesh and registration steps are shown in Figure 2.

Registrations were performed first on the coarse template to all the 43 aorta-only-geometries using Coherent Point Drift (CPD) (27, 28) using rigid, affine, and deformable transformations (α = 3, β = 15). After the initial registration of the coarse mesh, the mesh was subdivided using the subdivision matrices. A second deformable transformation (α = 3, β = 7) was performed to ensure the small details in the geometry were aligned properly and to correct the deflation effect of the subdivided surface. The two parameters α and β represent the trade-off between goodness of maximum likelihood fit and regularization, and the width of smoothing Gaussian filter, respectively (27).

Note that the coarse template is optional and was used to speed up the mesh registration process. Without a coarse template, the registration process would be performed directly using the normal template mesh with all 3 transformations (rigid, affine, and deformable) and no subdivision surface is necessary. However, different parameters for the deformable transformation may be required. The two step registrations were performed in this study to tune the parameters quickly on the coarse template mesh while ensuring they have sufficient registration accuracy for all geometries.

Finally, the registered surfaces were used to extract the wall shear stress vectors and magnitude from the CFD simulations. The spatial coordinates (x, y, z) of each mesh node were stored as a “flatmap” with 3 channels (1 for each axis), with the Cartesian coordinate system. The KDTree algorithm was used to obtain WSS vectors for every point on the registered surface by searching for the closest point in the CFD surface mesh, with a search radius of 5 mm from each surface node. Template nodes corresponding to the aortic branches were masked as “invalid” by applying a distance threshold of >1.2 mm radius. Manual inspection and corrections were performed subsequently to ensure other aortic surface regions were included, and only the aortic branches regions were invalid. Despite how the CFD simulations included branching vessels, the registered surfaces did not. On the base of the branching vessels on the registered surfaces, there were no actual WSS values, which renders these regions invalid. These invalid regions were not optimized during the loss calculation.

**Figure 3:**
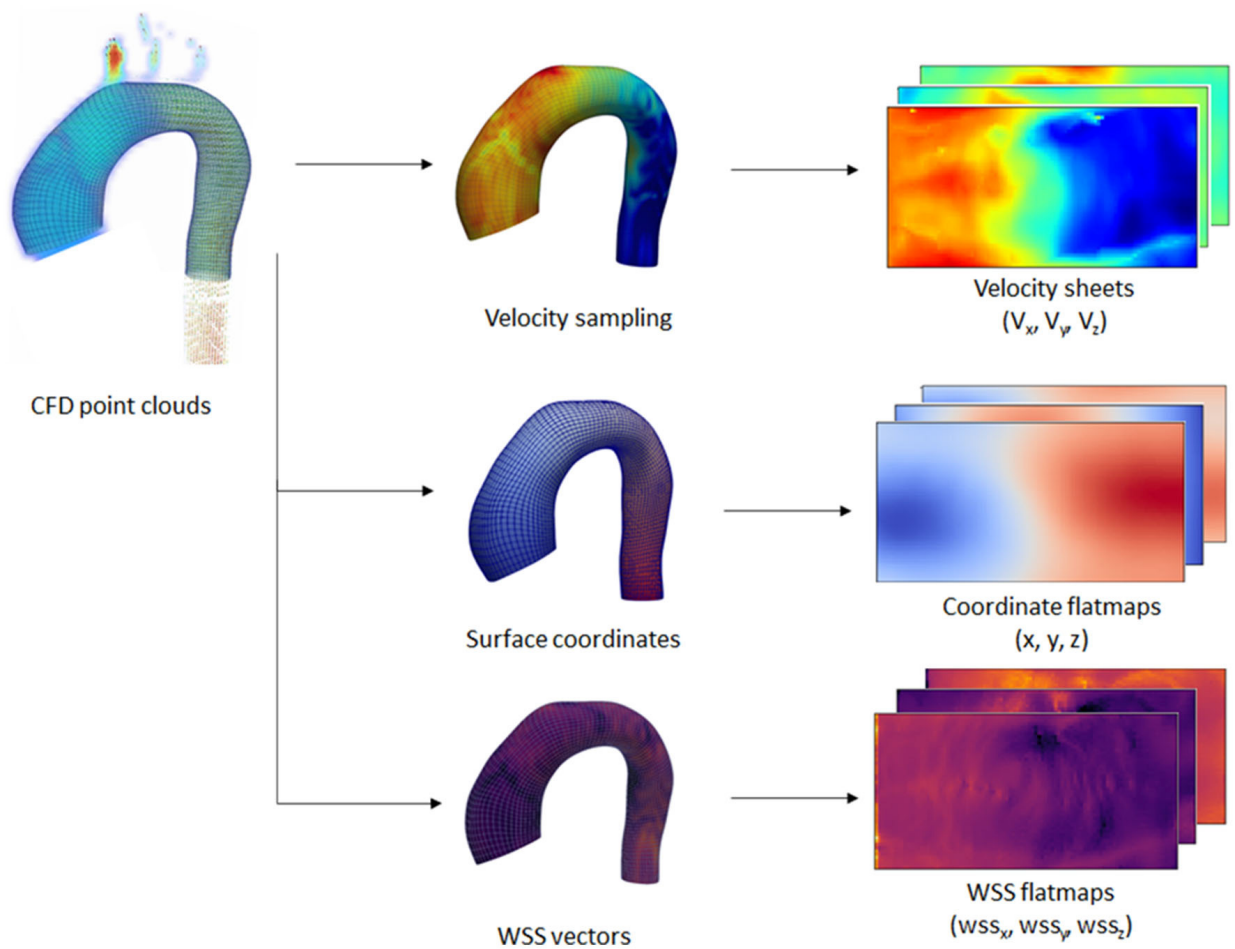
Overview of the extraction process performed on the CFD point clouds dataset. Extraction was performed on the wall coordinates and several inner surface coordinates. Wall shear stress (WSS) vectors and velocity vectors were extracted at the wall and inner coordinates, respectively. The extracted information was transformed into 2D flatmaps based on the template mesh.

Additionally, velocity vectors were extracted in varying inward distances (0.3, 0.5, 0.6, 0.8, 1.0, and 2.0 mm) normal to the surface points. Each velocity vector corresponded to each point with a predefined distance from the registered surface, forming a layer of velocity values, which we call a “velocity sheet.” Alongside this, the spatial coordinates of the internal surface were also stored as flatmaps. Due to the no-slip-wall boundary condition, the velocity sheet at the vessel surface was assumed to be 0 and, thus, was not extracted nor included as part of input data. The input data consisted of the registered surface mesh coordinates and the internal coordinates (points with variable inward distances normal from the surface) with their corresponding velocity vectors, while the ground truth label consisted of the 3D wall shear stress vectors at the registered surface mesh coordinates. An overview of the extracted information is shown in Figure 3.

### Network and Training

**Figure 4:**
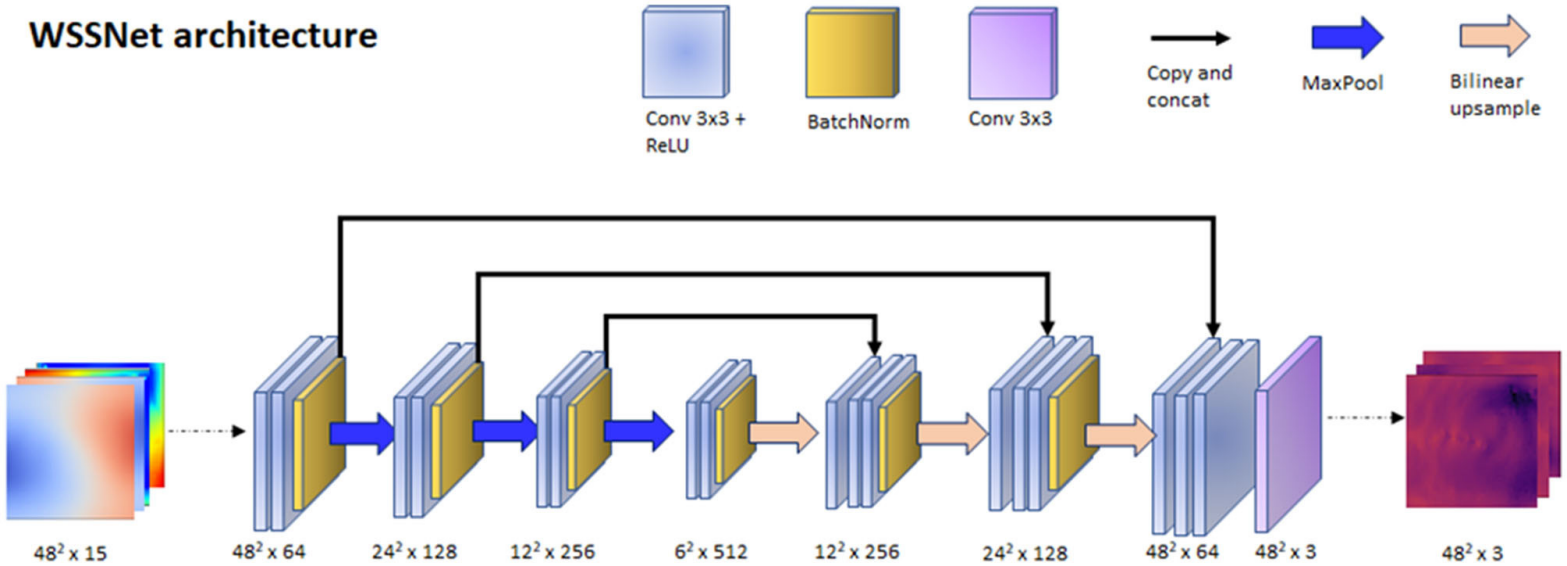
WSSNet architecture. The network is based on U-Net architecture, which receives an input of 15 channels of 48 × 48 patches, consisting of coordinate flatmaps and velocity sheets, and outputs Cartesian wall shear stress vector patches.

With the input and output data effectively represented as 2D images, we could leverage the convolutional neural network (CNN) capability in learning spatial relationships. The input of the network was a 15-channel tensor, consisting of the Cartesian wall coordinates (x_0_, y_0_, z_0_), two internal surface coordinates (x_1_, y_1_, z_1_, and x_2_, y_2_, z_2_), and two velocity sheets (v_x1_, v_y1_, v_z1_, and v_x2_, v_y2_, v_z2_). The output of the network is a 3-channel tensor, depicting the wall shear stress vectors (wss_x_, wss_y_, wss_z_). A U-Net-like structure was used for the network architecture. The network consisted of 3 encoder and decoder blocks, with each block consisting of 2 convolutional layers with Rectified Linear Unit (ReLU) activation function, followed by batch normalization at the end of the block. Max pooling was applied on each of the encoder blocks, while bilinear interpolation was utilized to upsample each of the decoder blocks. The network architecture is shown in Figure 4.

The network was trained using a patch-based approach, with a 48 × 48 patch, which matched the size of the template mesh's circumference. The patches were selected randomly through the length of the vessel, acting as a sliding window. To ensure the network learned about the circular nature of the patch, we introduced periodic/circular padding. This is done by padding the top-most row within the patch with the bottom-row and *vice-versa*, and by duplicating the value of the left or right most column in the longitudinal direction. Periodic padding was applied before the first two convolutional layers.

Several augmentation strategies were applied to the dataset:

1. Distance to wall: to ensure that the network learns the spatial features, v_1_ and v_2_ are a combination of the available sheets, with the v_1_ sheet closer to the wall than v_2_. The combination of different velocity sheets was random within a pre-defined range.
2. Translation: to simulate translation to the training dataset, we selected a random node within the wall coordinate patch, and subtract that node position from all the coordinates, effectively setting it as the origin.
3. Rotation: random 3D rotations on a randomly selected plane were applied to the coordinate flatmaps and velocity sheets.
4. Shift (sliding window): with the patch based approach, we shifted the patch in the longitudinal direction allowing the network to learn geometric and flow features on different regions of the vessel. This acts similar to the random selection of the patch.
5. Rolling-shift: with the cut-line of the template mesh predefined at the inner side of the aortic curve, the network might be fixated on the same geometric features (i.e., center rows having aortic branches). To introduce variation during the training, we perform a periodic-shift in the circumferential direction (U) by a maximum of 5 pixels.
6. Random noise: A 50% chance of adding Gaussian-smoothed Gaussian noise with an SD between 1 to 4% venc was added (venc = 1.5 m/s). The normally distributed noise was added to simulate the noise characteristics in the fluid domain. The Gaussian smoothing operation was added to simulate the interpolation that occurs when resampling CFD point clouds to a uniform grid.

**Figure 5:**
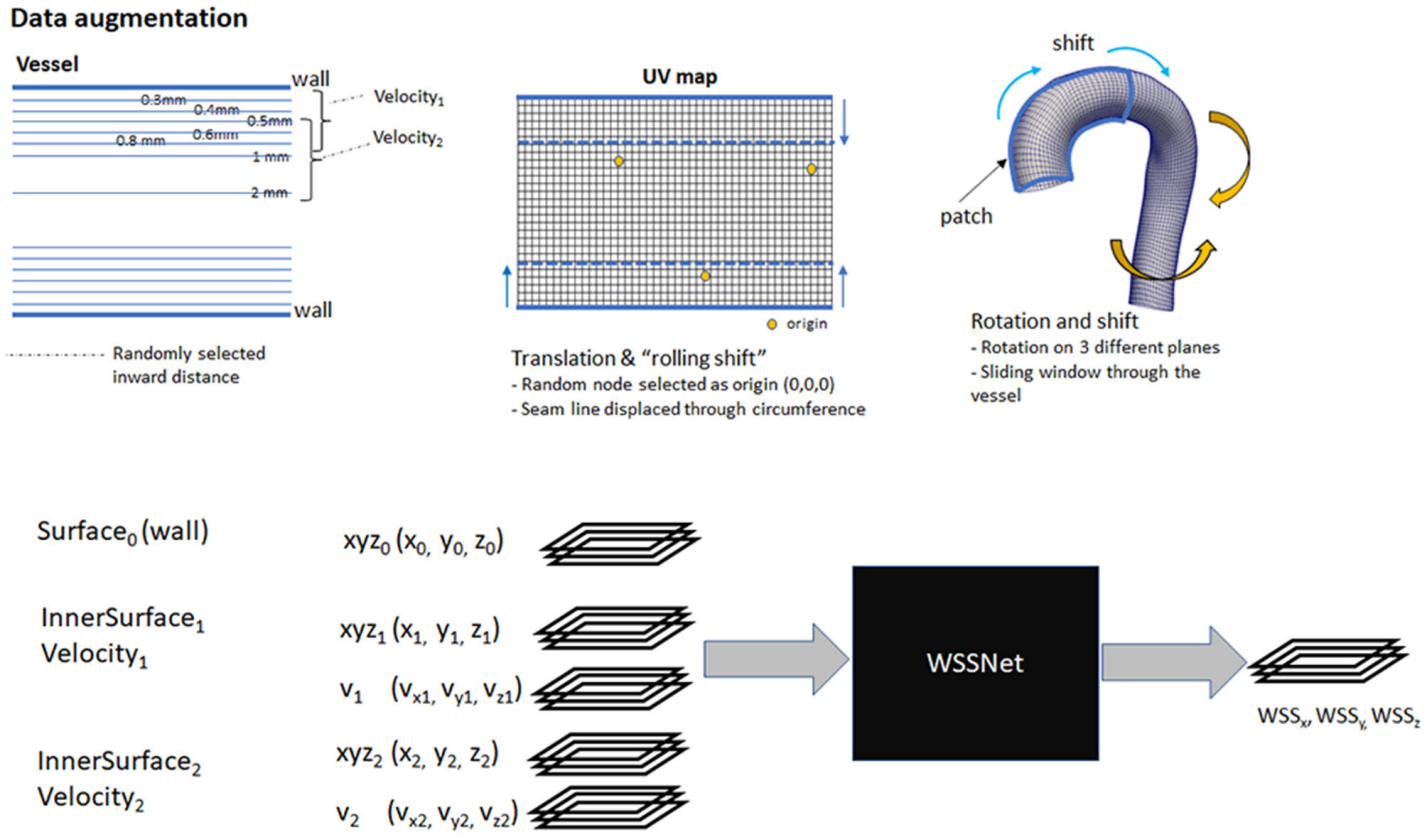
Top: Overview of augmentation strategies. Velocity sheets were extracted at various distances from the surface (0.3–2.0 mm). During training, two velocity sheets were chosen randomly, with the first one closer to the vessel than the other. Bottom: a global overview of the input and output of WSSNet. Input consists of 15 channels, consisting of 3 coordinate flatmaps and 2 velocity sheets. The output consists of 3 channels, correspond to wall shear stress vectors in Cartesian coordinates.

The first 3 pixels from the inlet were excluded during training to avoid overestimated WSS caused by CFD boundary values. Nodes outside the mask (the base of the aortic branches) were also excluded because the WSS obtained are not true WSS and are basically obtained from the aortic branches. Figure 5 summarizes the augmentation strategies, alongside a global overview of the network input and output.

The network was trained using Adam optimizer for 100 epochs, with a batch size (*m*) of 16. Cosine annealing learning rate was used on a repeating cycle for every 10 epochs, with a learning rate set between 10^−4^ and 10^−7^. Tensorflow 2.0 (29) was used as the backend of the training. The network was trained on a Titan X GPU with 16GB memory.

From the 43 CFD simulations, 37 simulations were used for training, 3 for validation, and 3 simulations reserved for testing. The datasets were split randomly, with 8 left ventricular hypertrophy cases ending up in the training set, 1 case in the test set. It is worth noting that the data generated using CFD simulations do not represent the actual *in vivo* measurements.

The training set consisted of flatmaps extracted directly from the CFD point clouds. To ensure that the network can generalize well to 4D Flow MRI data, the validation and test sets consisted of flatmaps extracted from the following data representations: (1) CFD point clouds, (2) downsampled 2.4 mm^3^ uniform grid (to mimic the MRI resolution), and (3) 2.4 mm^3^ grid with noise (normal distribution, SD of 2% venc to simulate 4DFlow data). More detailed explanations on the sampling process from CFD point clouds to simulate MRI are explained in the next section.

The training set comprised 46,676 unique flatmap combinations (mainly due to the combinations of velocity sheets with different distances), and the validation set consists of 3,996 unique flatmap combinations. Additionally, the sliding window strategy ensures the network “sees” different parts of the flatmap during the training process.

#### Loss Function Definition

A combination of loss functions was utilized, to ensure minimum difference of the WSS vectors and distributions (pattern similarity) between the predicted and reference values. First, we minimized the mean absolute error (MAE) between each of the predicted wall shear stress vector components and the reference values. Additionally, as we modeled the WSS flatmap as an image, we could optimize the pattern similarity between the predicted WSS magnitude and the ground truth WSS. Finally, an L2 regularization term was added to the network that can generalize to the new dataset, which was controlled by regularization weight (λ) scaled to the batch size (*m*). The complete loss function is given as


$$\begin{array}{l} loss=\;l_{MAE}+\omega \;l_{SSIM}+\frac{\lambda }{2m}\overset{N}{\underset{i=1}{\sum }}w_{i}^{2} \end{array}$$

with ω = 1.5 and λ = 10^−2^ to balance each of the loss terms to the same scale.

The Structural Similarity (SSIM) index, commonly used to measure the similarity of two images x and y, was added as a loss term to ensure WSS pattern similarity. SSIM is calculated based on three components: luminance (l), contrast (c), and structure (s). The luminance can be measured from the local average (μ) image values, while contrast is measured from the local SD (σ), and the structure index is measured using Pearson correlation (r).

Luminance comparison function *l*(*x, y*) can be defined as


$$\begin{array}{l} l\left(x,y\right)=\frac{2u_{x}u_{y}+C_{1}}{u_{x}^{2}+\;u_{y}^{2}+\;C_{1}} \end{array}$$

while contrast comparison function *c*(*x, y*) is defined by


$$\begin{array}{l} c\left(x,y\right)=\frac{2\sigma_{x}\sigma_{y}+C_{2}}{\sigma_{x}^{2}+\;\sigma_{y}^{2}+\;C_{2}} \end{array}$$

and structural comparison function *s*(*x, y*) is used to measure the linear correlation between the two images:


$$\begin{array}{l} s\left(x,y\right)=r\;=\frac{\sigma_{xy}+C_{3}}{\sigma_{x}\sigma_{y}+\;C_{3}} \end{array}$$

with σ_*xy*_ being the covariance of the two images, denoted as


$$\begin{array}{l} \sigma_{xy}=\frac{1}{n}\;\overset{n}{\underset{i=1}{\sum }}\left(x_{i}-u_{x}\right)\left(y_{i}-u_{y}\right) \end{array}$$

C_1_, C_2_, and C_3_ are constants added for numerical stability. C_1_ = (K_1_ L)^2^, C_2_ = (K_2_ L)^2^, and C_3_ = C_2_/2 are defined with K_1_ = 0.01 and K_2_ = 0.03 as in the original article (30), with L being the maximum true WSS within a patch.

Overall, SSIM is a combination of all the terms above:


$$\begin{array}{l} SSIM\left(x,y\right)=l{\left(x,y\right)}^{\alpha }\;.\;c{\left(x,y\right)}^{\beta }\;.\;s{\left(x,y\right)}^{\gamma } \end{array}$$

where α, β, and γ are positive numbers, denoting the relevance of each term, with α = β = γ = 1. With that definition, SSIM loss is described as


$$\begin{array}{l} l_{SSIM}=1-SSIM\left(x,y\right) \end{array}$$

A built-in SSIM implementation from Tensorflow was used for the training process, with the default local region of 11 × 11 pixels.

### Evaluation

Overall, evaluation of the network was performed in three different stages:

1. Evaluation on CFD simulation data (point cloud data) The network was validated on 6 CFD simulations with each of 72-time frames (*n* = 432). The input flatmaps were extracted directly from CFD point clouds.
2. Evaluation of CFD simulation data (synthetic MRI grid) The network was validated on 6 CFD simulations with each of 20 time frames (*n* = 120). The CFD point clouds were first interpolated into 3D grid representation (synthetic MRI) before extracting the flatmaps. The network was validated on two different grid resolutions (2.4 and 1.2 mm isotropic) and with/without noise, resulting in four sets of validations.
3. Evaluation on *in vivo* 4D Flow MRI The network was validated on all 43 *in vivo* cases, with each of 20 time frames (*n* = 860). These 43 cases were utilized previously for the aortic geometry extraction only. The flow information obtained from these *in vivo* cases does not resemble the generated CFD simulations.

#### Evaluation Metrics

For quantitative assessment, performance was evaluated with respect to the difference in point-to-point WSS magnitude per time frame, reported as mean absolute error (MAE) and relative error. Relative error was calculated as the ratio of absolute WSS difference and peak reference WSS value at the specified time frame. In addition, Pearson correlation (*r*) was also reported to evaluate the pattern similarity or WSS distribution for every time frame.

For quantitative and qualitative assessment, TAWSS and OSI were also calculated at each point in the template. TAWSS represents the average WSS over a cardiac cycle, while OSI represents the oscillation of the WSS direction over a cardiac cycle, computed as follows:


$$\begin{array}{l} TAWSS=\;\frac{1}{T}\int_{0}^{T}|\;\vec{wss\;}|\;dt\;\\ \;OSI=0.5\;\times \;\left(1\;-\frac{\left|\int_{0}^{T}\vec{wss\;}dt\right|}{\int_{0}^{T}|\;\vec{wss\;}|\;dt}\right) \end{array}$$

where T is the span of time for a cardiac cycle. OSI ranges from 0 to 0.5, with 0 describing no change in WSS during a cardiac cycle, and 0.5 where there is a change of direction of 180^o^ during a cardiac cycle. Additionally, a single-measure intraclass correlation coefficient (ICC) based on a two-way mixed-effects model was independently calculated for each measured TAWSS value to assess the degree of absolute agreement between WSSNet and ground truth CFD.

#### Surface Extraction Error

The registration step was performed to align and deform the template mesh to the aorta-only geometry, which is not without error. This imprecision of the vessel wall causes the inward points where velocity sheets were extracted to not be at the exact distance from the wall. While this imprecision introduces error to the training data, it also simulates actual inaccuracy in segmentation, where it is hampered by spatial resolution and partial volume effects. To measure the inaccuracy of the mesh registration step, we evaluated the surface distance for every node in the template mesh.

#### Validation on CFD Point Clouds

To evaluate the performance of WSSNet to reproduce CFD WSS estimations, the evaluation metrics were computed using the velocity data obtained directly from the CFD point clouds, on the 3 validation and 3 test cases, with each case consisting of 72 time frames (dt = 10 ms). For this validation, we evaluate the results by measuring MAE, relative error, Pearson correlation, TAWSS, and OSI compared to the ground truth WSS from CFD.

Finally, linear regression analysis was performed against reference values derived from CFD, assessing TAWSS and OSI, separately. Bland-Altman plots of the same data were also extracted to assess potential network bias. ICC was evaluated to assess the absolute agreement between the TAWSS values from the network against the ground truth CFD.

#### Validation on Synthetic MRI From CFD Point Clouds

To evaluate the capability of the network in predicting WSS from MR image resolution data, we first sampled the point cloud CFD dataset into a voxelized uniform image grid. To match the commonly acquired MRI spatial resolution, data were generated with isotropic spatial sampling of dx = 2.4. Furthermore, spatial sampling of dx = 1.2 mm was also performed to see how the network performs in different resolutions. Both resolutions were sampled at temporal resolution (dt) of 40 ms, within the range of common MRI acquisition. Additionally, noise (normal distribution, the SD set to 2% of the venc; venc = 1.5 m/s) was added to evaluate the performance of the network in the presence of noise. Thus, the evaluation set consisted of noise-free and noisy data at both resolutions.

Afterward, we performed the same procedures to extract the coordinate flatmaps and interpolate the velocity sheets from the synthetic MRI grid. Velocity sheets were extracted using linear interpolation at 1.0 and 2.0 mm at the inward direction normal to the surface. Subsequently, these coordinate flatmaps and velocity sheets were then used as input to the network.

As a comparison, the parabolic fitting method (9, 11) was selected as it is commonly employed and requires similar input. The method requires 3 velocity points, where the velocity at the wall is assumed as 0, due to the no-slip boundary condition. With the given resolution (dx = 2.4 and 1.2 mm), the parabolic fitting method is expected to underestimate the WSS values. For this validation, we performed the same quantification with the CFD point clouds dataset (MAE, relative error, Pearson correlation, TAWSS, and OSI) because the ground truth WSS was known.

Linear regression analysis was performed for both methods against reference values derived from CFD, assessing TAWSS and OSI at different resolutions: noise-free 2.4 mm, noisy 2.4 mm, noise-free 1.2 mm, and noisy 1.2 mm. Bland-Altman plots were extracted to assess potential network bias for the noisy 2.4 mm dataset. Additionally, Bland-Altman plots were extracted to assess the agreement between the parabolic fitting method and WSSNet predictions for the noisy 2.4 mm dataset. For each of the resolutions, ICC was evaluated for both methods to assess the absolute agreement between the TAWSS values from the network against the ground truth CFD.

#### Validation on *in vivo* Cases

**Figure 6:**
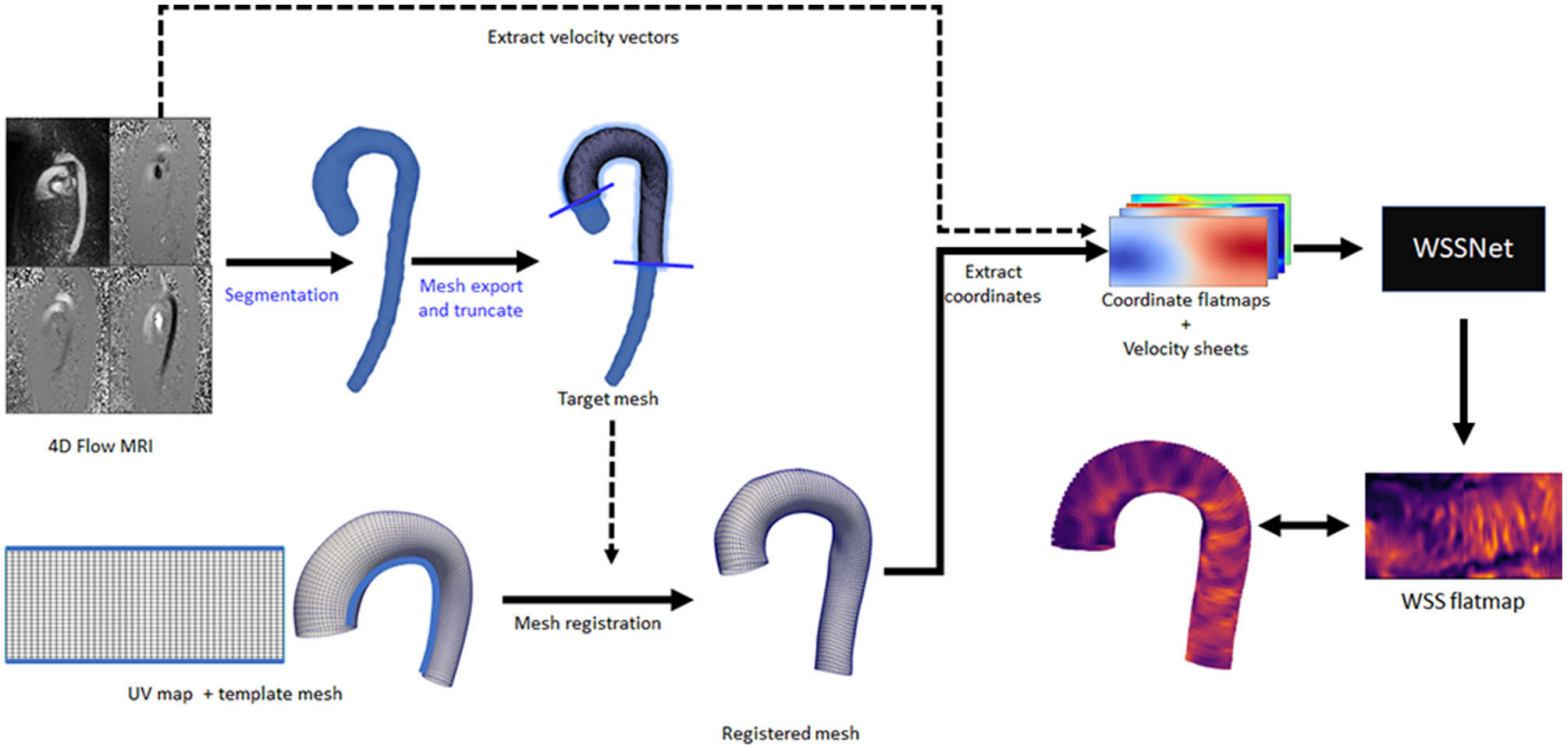
Complete overview of the inference workflow for 4D Flow MRI. All the steps are fully automated, except for the segmentation and mesh truncation steps (marked with blue text).

To assess WSS on *in vivo* data, the method was applied to the *in vivo* 4D Flow data from the same 43 cases used in the patient specific CFD simulations. The same registered surface meshes were used to extract the coordinate flatmaps and velocity sheets. Velocity sheets were extracted with inward distances of 1.0 and 2.0 mm due to the inherent MRI resolution at approximately dx = 2.4 mm. Figure 6 shows an overview of the analysis pipeline for 4D Flow MRI to WSS flatmap.

For these *in vivo* cases, the WSS reference values are not available. Due to the expected difference in WSS magnitudes (between WSSNet and parabolic fitting), only Pearson correlation was evaluated between the two methods. In addition, TAWSS and OSI were also visualized.

Linear regression analysis was performed against WSSNet predictions as reference values, assessing TAWSS and OSI, separately. Bland-Altman plots of the same data were also extracted to assess bias between the two methods.

## Results

### Surface Extraction Error

The WSS ground truth was obtained from the CFD point closest to the registered template mesh nodes. Surface distance errors for the aortic vessel wall were 0.32 ± 0.14 mm, rising to 2.38 ± 1.08 mm at the base of the aortic branches where there was no true wall. Nevertheless, the error was small relative to the current MRI resolution (2.4 mm).

### Validation on CFD Point Cloud Dataset

**Figure 7:**
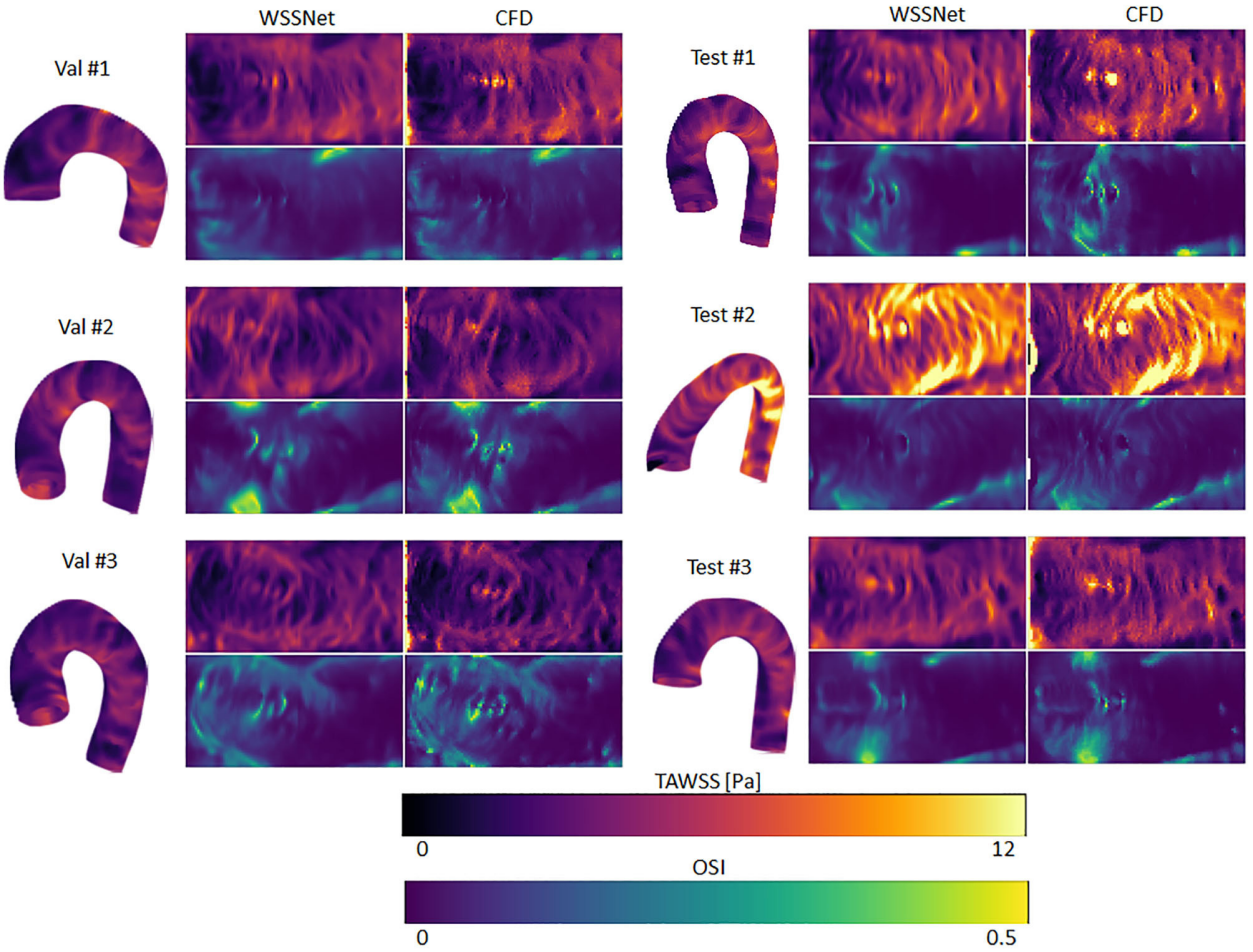
Time-averaged WSS and oscillatory shear index (OSI) comparison between WSSNet and ground truth CFD. Time-averaged WSS (TAWSS) and OSI were calculated from all time frames (n = 72). 3D representations of the TAWSS from WSSNet are shown on the left side of each flatmap.

A complete evaluation was performed on the 3 validation and 3 test cases, with each case consisting of 72 time frames (dt = 10 ms). Overall, WSS estimates were accurate (MAE 0.55 ± 0.60 Pa, relative error 4.34 ± 4.14%) and showed excellent Pearson correlation with CFD WSS (0.92 ± 0.05). More detailed quantitative measures per case are given in Table 1. Figure 7 shows the qualitative results for each of the cases, represented by the TAWSS and OSI.

**Table 1:** Wall shear stress (WSS) magnitude and pattern similarity measurements for the validation and test cases from the CFD simulations.

**Case (characteristics)**	**MAE (Pa)**	**Rel error (%)**	**Pearson correlation**
Val #1 (normal)	0.44 ± 0.41	5.97 ± 4.82	0.92 ± 0.06
Val #2 (normal)	0.44 ± 0.50	3.70 ± 4.03	0.93 ± 0.03
Val #3 (normal)	0.49 ± 0.43	4.67 ± 4.22	0.92 ± 0.02
Test #1 (normal)	0.57 ± 0.75	2.87 ± 3.43	0.91 ± 0.04
Test #2 (LVH)	0.93 ± 1.04	5.76 ± 5.24	0.88 ± 0.09
Test #3 (normal)	0.44 ± 0.48	3.09 ± 3.12	0.94 ± 0.03
Overall	**0.55** **±0.60**	**4.34** **±4.14**	**0.92** **±0.05**

*For each case, results were measured from all time frames (n = 72, dt = 10 ms). MAE, mean absolute error; LVH, left ventricular hypertrophy. Average values are shown in bold*.

**Figure 8:**
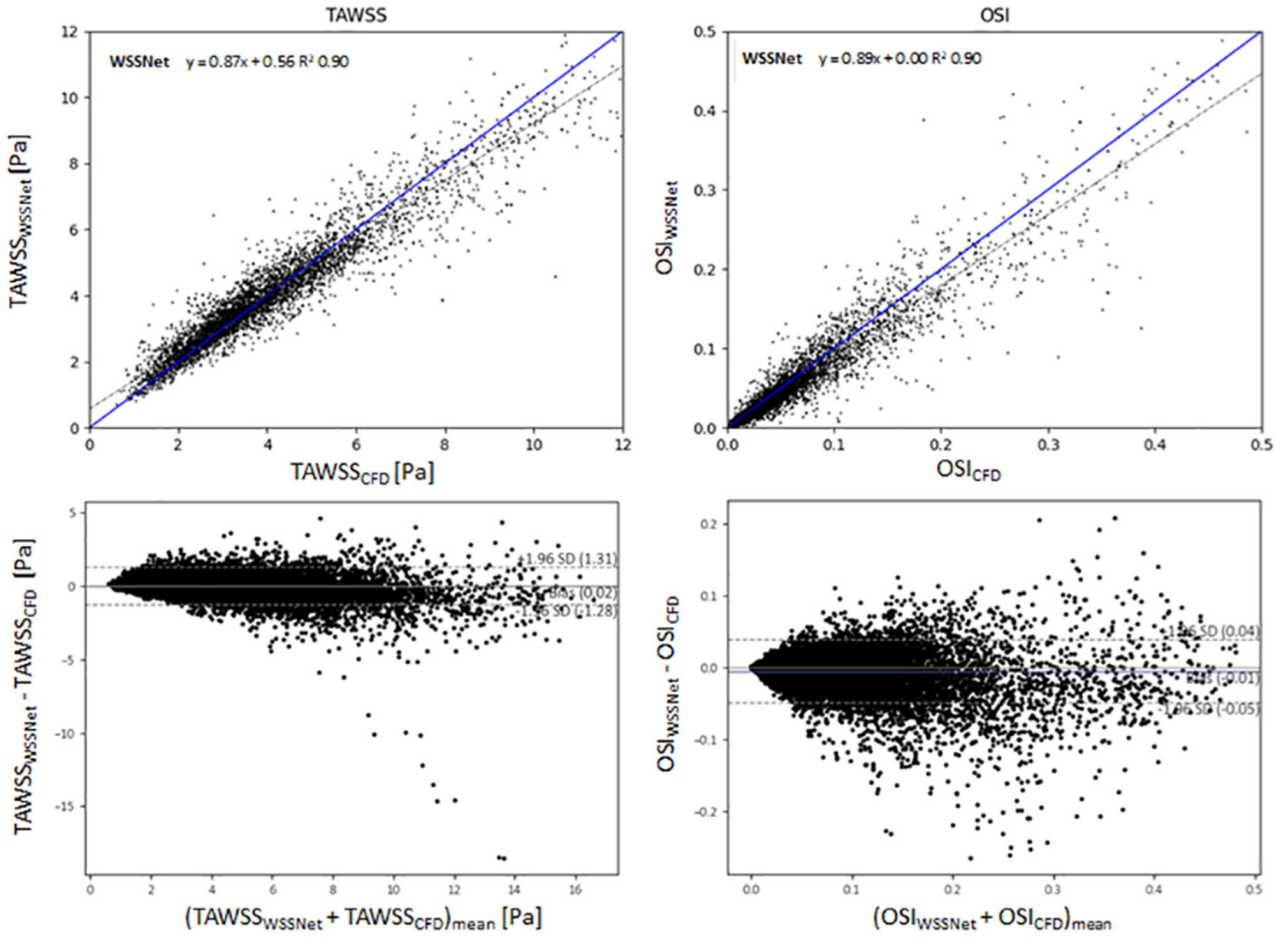
Top: Regression plot for TAWSS and OSI between estimated values from WSSNet and ground truth CFD. TAWSS and OSI have computed over 6 cases (3 validation and 3 test) averaged over 72 time frames (dt = 10 ms). Bottom: Bland-Altman plots for TAWSS and OSI. The plots show a point-wise comparison within the flatmap. The plots show 20% of the data points, randomly selected.

Figure 8 shows linear regression plots and Bland-Altman representations for the calculated TAWSS and OSI. In general, very high correlations are observed between TAWSS and OSI estimated by WSSNet and ground truth CFD, with linear regression slopes and coefficients of determination of k = 0.88 and R^2^ = 0.90, and k = 0.88 and R^2^ = 0.91 for TAWSS and OSI, respectively. Bland-Altman analysis shows a minimal bias (0.08 Pa) and limits of agreement of ± 1.29 Pa for TAWSS, and no bias for OSI with limits of agreement of ± 0.04. ICC shows excellent agreement (0.95) between TAWSS calculated from WSSNet and ground truth CFD.

### Validation on CFD Synthetic MRI Dataset

Velocity sheets were extracted from the synthetic MRI dataset on the same 6 validation/test cases. Compared with the prediction on the sheets extracted from the CFD point clouds, a decrease in performance was observed when inference was performed on dx = 2.4 mm (MAE 0.94 ± 0.87 Pa), while Pearson correlation was still highly maintained (r = 0.82 ± 0.08). The addition of noise slightly decreased the performance further (MAE 0.99 ± 0.91 Pa, r = 0.79 ± 0.10). ICC showed good agreement with similar values, 0.86 and 0.85, for noise-free and noisy data, respectively.

At twice the resolution (dx = 1.2 mm), WSSNet showed better performance in predicting WSS (MAE = 0.65 ± 0.67 Pa, r = 0.89 ± 0.06) compared to the base resolution (dx = 2.4 mm). The addition of noise at 1.2 mm resolution impacted the performance slightly (MAE = 0.71 ± 0.71 Pa, r = 0.85 ± 0.10), showing reduced error and Pearson correlation. ICC went back up to 0.92 for both noise-free and noisy data at 1.2 mm resolution, getting closer to the CFD validation counterpart (0.95).

On the other hand, the parabolic fitting method showed much larger differences (MAE 2.89 ± 1.85 Pa and 2.33 ± 1.67 Pa at dx = 2.4 mm and 1.2 mm, respectively). The values were underestimated, mostly at regions of peak WSS. In terms of Pearson correlation, the parabolic fitting results showed moderate correlation with the CFD ground truth (r = 0.65 ± 0.12 and 0.69 ± 0.11, at dx = 2.4 and 1.2 mm, respectively).

**Figure 9:**
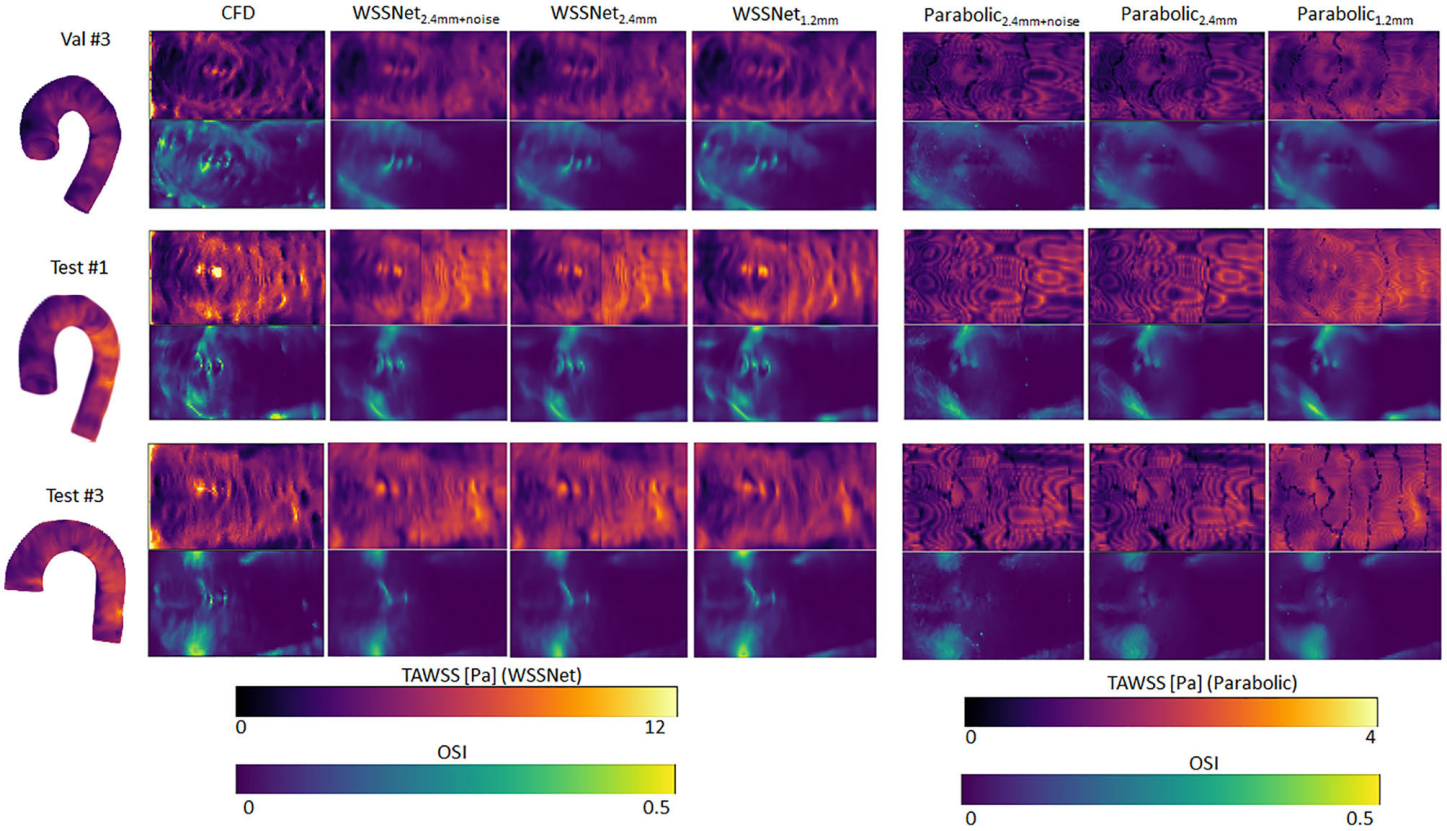
Time-averaged WSS and OSI comparison between WSSNet and parabolic fitting method at a different spatial resolution of synthetic MRI (dx = 2.4 mm, 2.4 mm with noise and 1.2 mm). For reference, TAWSS and OSI flatmaps from ground truth CFD are provided in the left-most column. TAWSS derived from the parabolic method were much lower and showed different dynamic ranges (0–4 Pa) to highlight pattern similarity between methods. 3D representations of the TAWSS from WSSNet_2.4+noise_ are shown on the left side of each flatmap.

Qualitative assessments are shown in Figure 9, represented as TAWSS and OSI. WSSNet predictions show good pattern similarity at both resolutions, with less detail recovered at dx = 2.4 mm. The parabolic fitting method showed WSS magnitude roughly 3 times lower than the CFD magnitude. For both algorithms, the OSI pattern appeared similar at both resolutions. Table 2 shows a complete overview of the metrics for both methods on different resolutions.

**Table 2:** WSS magnitude and pattern similarity measurements for the validation and test cases of the CFD simulations.

**Method (resolution)**	**MAE (Pa)**	**Rel error (%)**	**Pearson correlation**	**ICC(A,1)**
WSSNet_CFD_	**0.55** **±0.60**	**4.34** **±4.14**	**0.92** **±0.05**	**0.95**
WSSNet_2.4mm_	0.94 ± 0.87	6.35 ± 5.67	0.82 ± 0.08	0.86
WSSNet_2.4mm+noise_	0.99 ± 0.91	7.13 ± 6.27	0.79 ± 0.10	0.85
WSSNet_1.2mm_	0.65 ± 0.67	4.80 ± 4.47	0.89 ± 0.06	0.92
WSSNet_1.2mm+noise_	0.71 ± 0.71	5.66 ± 5.13	0.85 ± 0.10	0.92
Parabolic_2.4mm_	2.89 ± 1.85	14.63 ± 6.83	0.65 ± 0.12	0.09
Parabolic_2.4mm+noise_	2.89 ± 1.85	14.63 ± 10.69	0.59 ± 0.11	0.09
Parabolic_1.2mm_	2.33 ± 1.67	11.23 ± 5.82	0.69 ± 0.11	0.13
Parabolic_1.2mm+noise_	2.65 ± 1.75	13.07 ± 9.76	0.68 ± 0.11	0.13

*The table shows the comparison of WSSNet and parabolic fitting on different spatial resolutions (dx = 2.4 and 1.2 mm isotropic) with and without noise (n = 120 each). The result from the CFD point clouds (WSSNet_CFD_) is included for comparison (n = 432). Intra-class correlation (ICC) is computed from time-averaged WSS. Baseline values (WSSNet on CFD data) are shown in bold*.

**Figure 10:**
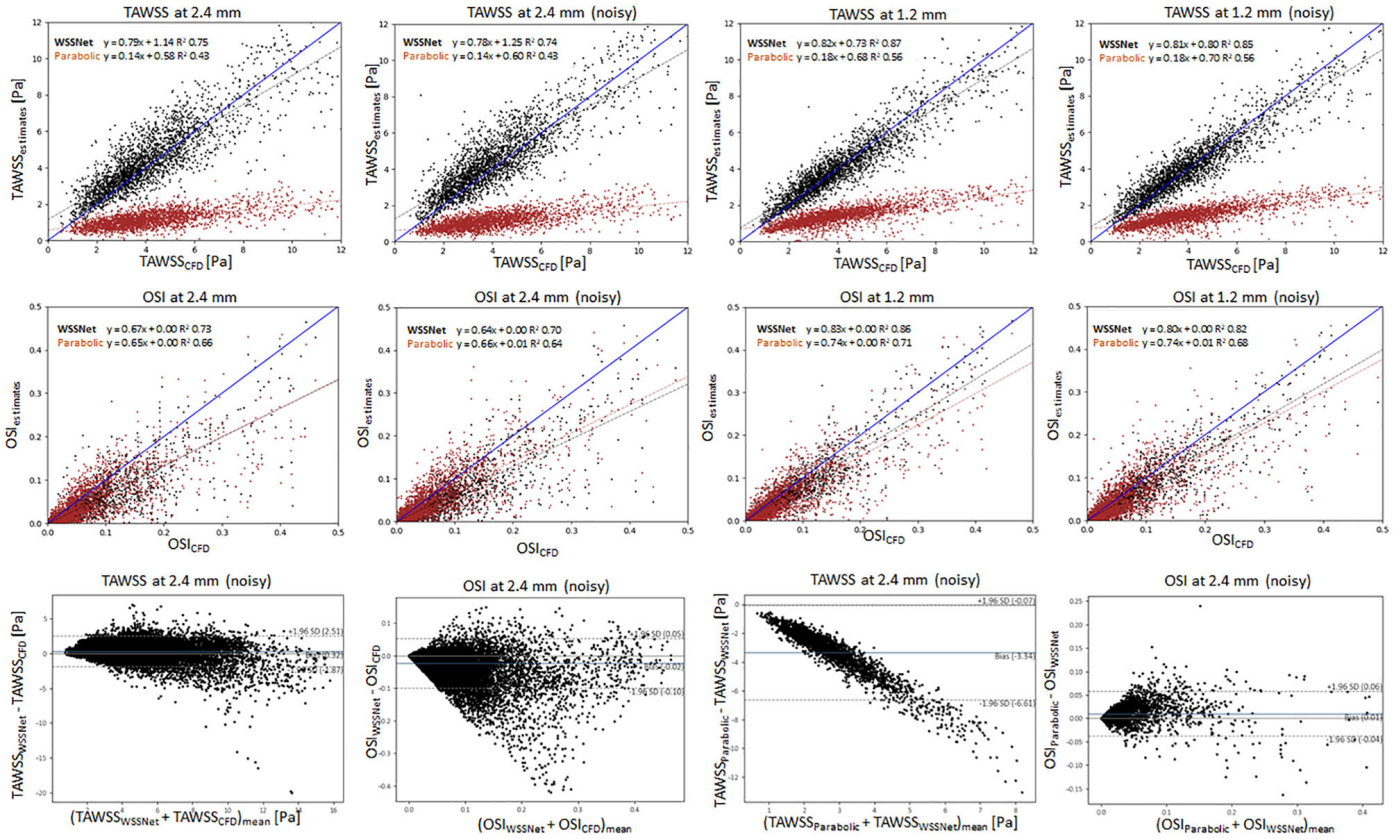
Top: Linear regression plots of TAWSS for synthetic MRI using WSSNet (black) and parabolic fitting method (brown) at different resolutions (dx = 2.4 and 1.2 mm) with and without noise, compared with ground truth CFD. Middle: linear regression plots for OSI at different resolutions (dx = 2.4 and 1.2 mm) with and without noise, compared with ground truth CFD. Bottom left: Bland-Altman plots of TAWSS and OSI between WSSNet and ground truth CFD at 2.4 mm noisy synthetic MRI. Bottom right: Bland-Altman plots of TAWSS and OSI between parabolic fitting method and WSSNet at noisy 2.4 mm synthetic MRI. The plots show 20% of the data points, randomly selected.

Figure 10 shows linear regression plots and Bland-Altman representations for both methods. In general, good correlations are observed for TAWSS between WSSNet and CFD ground truth, with linear regression slopes and coefficients of determination of k = 0.79 and R^2^ = 0.75 reported for the noise-free 2.4 mm resolution, and k = 0.78 and R^2^ = 0.74 for the noisy 2.4 mm resolution. Slightly higher values are seen for WSSNet at 1.2 mm (k = 0.82 and R^2^ = 0.87 for noise-free; k = 0.81 and R^2^ = 0.85 for noisy). As a comparison, the parabolic fitting method shows poor correlations with CFD ground truth (k = 0.14 and R^2^ = 0.43 for noise-free and noisy 2.4 mm; k = 0.18 and R^2^ = 0.56 for noise-free and noisy 1.2 mm).

In terms of OSI, WSSNet shows moderate correlation at 2.4 mm (k = 0.67 and R^2^ = 0.73 for noise-free; k = 0.64 and R^2^ = 0.70 for noisy) and good correlation at 1.2 mm (k = 0.83 and R^2^ = 0.86 for noise-free; k = 0.80 and R^2^ = 0.82 for noisy). Similarly, the parabolic fitting method shows moderate correlation at 2.4 mm (k = 0.65, R^2^ = 0.66 for noise-free; k = 0.66 and R^2^ = 0.64 for noisy) and slightly better correlation at 1.2 mm (k = 0.74, R^2^ = 0.71 for noise-free; k = 0.74, R^2^ = 0.68 for noisy).

Bland-Altman plots were assessed at noisy 2.4 mm resolution to show the quality of results at common MRI resolution without any preprocessing. Bland-Altman plot indicated minimal TAWSS bias (0.32 Pa) with limits of agreement of 2.19 Pa between WSSNet and reference CFD. For OSI, the Bland Altman plot indicated minimal bias (−0.02) with limits of agreement of 0.08.

To compare the agreement between the parabolic fitting method and WSSNet, the Bland-Altman plot was also assessed at noisy 2.4 mm resolution. For TAWSS, Bland-Altman indicated a bias of −3.34 Pa, with higher TAWSS values showing larger differences (underestimation) by parabolic fitting method than WSSNet. Conversely, the Bland-Altman plot for OSI shows only a minimal bias (0.01) with narrow limits of agreement (0.05).

### Validation on *in vivo* 4D Flow MRI Cases

For the *in vivo* dataset, validation was performed on all 43 cases at the base resolution (2.375 mm x 2.375 × 2.75 mm) as is without any denoising. WSS was computed for all the cases with both the parabolic fitting method and WSSNet at every time frame. The resulting TAWSS and OSI were then compared between both methods.

**Figure 11:**
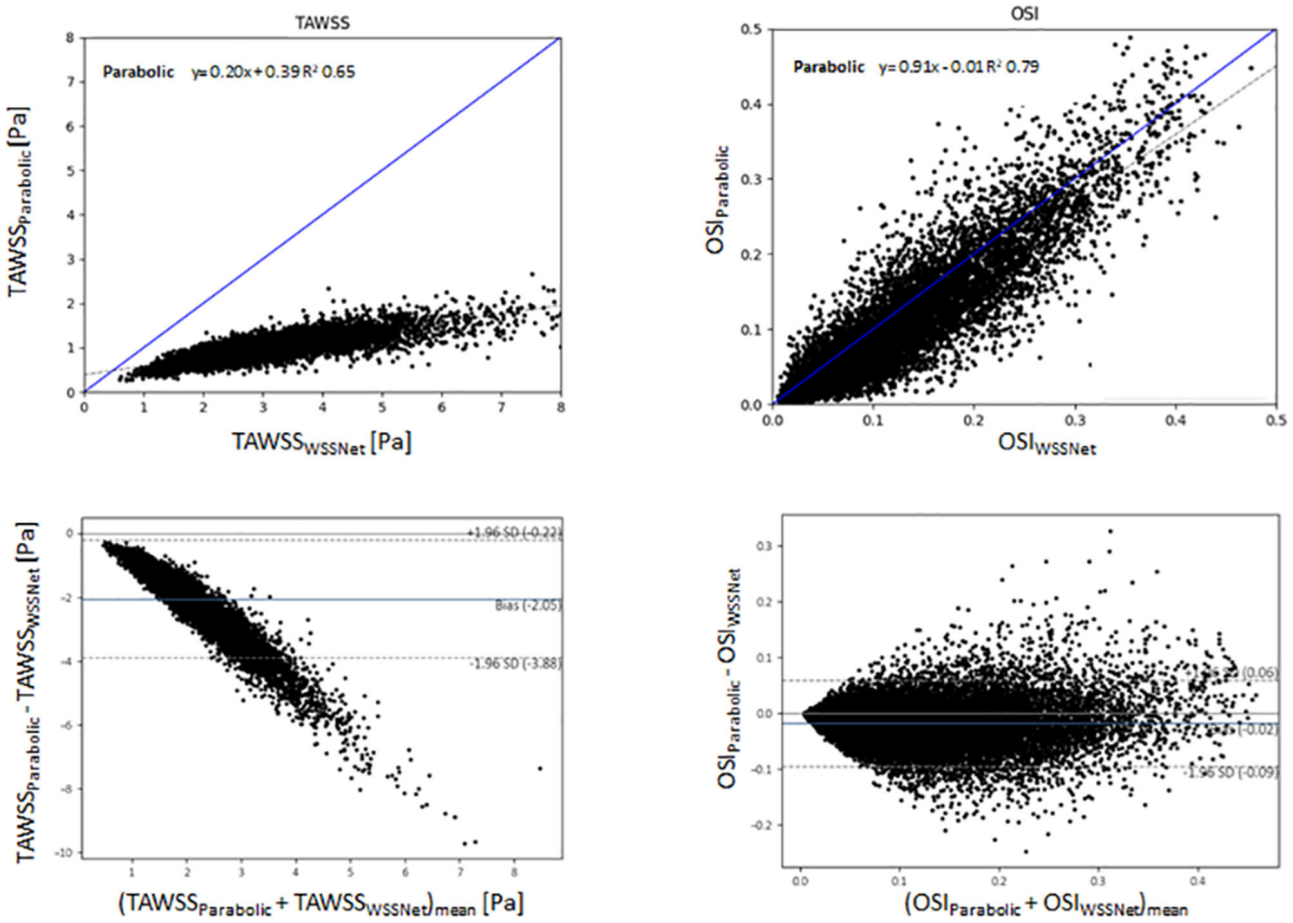
Top: Linear regression comparing the TAWSS and OSI derived from the estimation of WSSNet and parabolic fitting method from *in vivo* 4D Flow MRI. WSSNet estimates are used as the reference values. Bottom: Bland-Altman plots of TAWSS and OSI between the parabolic fitting method and WSSNet for *in vivo* cases (n = 43). The plots show 5% of the data points, randomly selected.

Figure 11 shows linear regression and Bland-Altman plots for TAWSS and OSI comparing both methods, using WSSNet predictions as reference values. Relative to WSSNet, the parabolic fitting method shows poor correlation (k = 0.20 and R^2^ = 0.65) for TAWSS but excellent OSI correlation (k = 0.91 and R^2^ = 0.71). Bland-Altman plot of TAWSS shows a bias of −2.05 Pa with a similar downward trend observed in the synthetic MRI, with higher TAWSS showing more underestimations. Conversely, the Bland-Altman plot of OSI shows minimal bias (−0.02) with limits of agreement <0.08.

**Figure 12:**
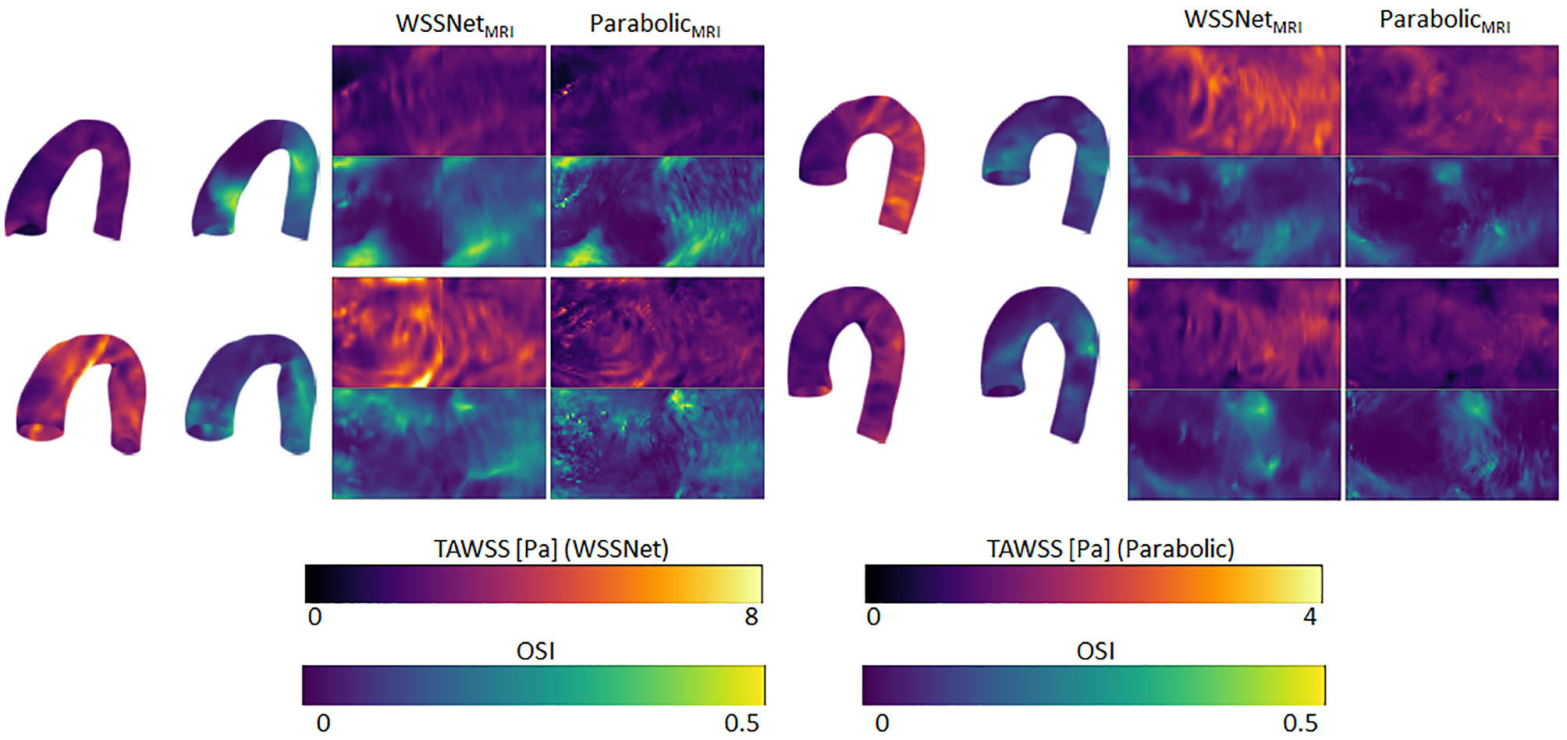
Time-averaged WSS and OSI comparison between WSSNet and parabolic fitting method in 4 cases of *in vivo* 4D Flow MRI. TAWSS derived from the parabolic method were much lower and shown using different dynamic ranges (0–4 Pa) to highlight pattern similarity between methods 3D representations of the TAWSS and OSI from WSSNet are shown on the left side of each flatmap.

Figure 12 shows visual comparisons of some of the cases using both methods. Visual inspection confirms the similarity between the computed TAWSS pattern, even though a clear difference in magnitude can be seen from the visualization. The spatiotemporal average WSS was 2.95 ± 1.57 Pa and 0.95 ± 0.46 Pa, for WSSNet and parabolic fitting method, respectively. Visual pattern similarity in OSI between both methods can be seen, which was observed in the synthetic MRI cases before. To quantify the similarity between WSS patterns, Pearson correlation was computed at every time frame, resulting in 0.68 ± 0.12.

## Discussion

This study demonstrates the feasibility of estimating WSS from low-resolution 4D Flow MR images using a deep learning method trained on a synthetic dataset acquired from CFD. Inference speed was 9 frames per second (26 cases per min) on a CPU for a typical 4D Flow MR image. The preprocessing step, which consisted of mesh registration and velocity sheet extraction, took ~10 min. Currently, aortic curve segmentation remains a manual process, which can be improved in the future. This workflow is several orders of magnitude faster than computational simulations while still offering good accuracy, which is not attainable using conventional methods at standard MRI resolution.

### Spatial and Velocity Informed Neural Network

Previous studies have shown neural network's capability to estimate hemodynamic variables from geometric features (15–18). While these methods were able to estimate hemodynamic variables with sufficient accuracy, the estimated values produced were time-averaged or specific to a static boundary condition, as no other quantities (i.e., pressure, velocity) were provided as input values. Consequently, these methods learned strictly from spatial features and were agnostic to flow. In practice, clinical data contains temporal information, for instance, flow velocity, which can be used to derive other hemodynamic variables, such as pressure and WSS.

The aortic template mesh utilized in this study is based on Liang et al. (18) which consisted of 5,000 nodes, which when unwrapped into a UV map, becomes a 50 × 100 quadrilateral mesh. We adapted the template into a 48 × 93 grid to accommodate the circumferential direction in fitting the U-Net input size (48 × 48) and the longitudinal direction to enable compatibility with applying subdivision matrices on the coarse template (12 × 24). The subdivision matrices are equivalent to applying subdivision surfaces two times, which increased the number of nodes to 2*n* and 2*n*-1 in a single pass, for circumferential and longitudinal direction, respectively. While the size of the circumference is fixed (48), different templates with different longitudinal axis lengths can be used (i.e., 48 × N template). As previously mentioned, the coarse mesh and subdivision surfaces were optional and were used to speed up the registration process. Because the network was trained on a patch-basis, a template that contains an extension of the aorta (e.g., more distal part of the descending aorta) may still be predicted by the network, and simply assumed as another patch. With this approach, the network is not fixated on specific markers across the aortic vessels but is more general in predicting WSS, as long as the vessel can be unwrapped into a UV map with the specified circumference size (U = 48).

In this study, we extended on these previous works by adding velocity sheets and coordinate flatmaps, which are crucial to calculate spatial velocity gradients at the vessel wall. We were motivated by previous widely used velocity-based methods, such as the linear extrapolation method, velocity-based-with-wall-position method, and the parabolic fitting method (11, 31). The idea of these methods is to calculate spatial velocity gradients from several inward distance velocities normal to the wall. Potters et al. (10) further implemented the velocity-based method using spline fitting for volumetric image with a similar approach using 3 or more velocity points (including wall point, which is assumed to be 0). While it is possible to use more than 3 points, Potters et al. showed that using 3 points resulted in more accuracy, given enough voxels were available across the diameter.

To ensure the network learns different distances of velocity sheets, the training data contained various predefined distances from the wall surface. To mitigate zero values at the velocity sheets caused by the registration error, the first velocity sheets were extracted at a 0.3 mm inward distance from the surface.

From the aforementioned studies, spatial hemodynamic variables (i.e., WSS, ECAP, pressure) can be derived from geometric features alone. Conversely, conventional velocity-based methods are already used for *in vivo* cases resulting in spatiotemporal WSS. By combining these two concepts, we were able to train a network capable of estimating spatiotemporal WSS more accurately, by using geometry and velocity information.

It is noteworthy that WSSNet returns the WSS vectors at the vessel surface, which is useful for deriving other variables, such as TAWSS, OSI, and different WSS components (i.e., circumferential and longitudinal WSS). Previous studies explored the importance of this directional WSS (2, 32) and WSS angle (33). Increased axial WSS can be an indicator of the presence of high-risk plaque (32) and another study suggested that axial WSS might explain different morphologies in ascending aorta dilatation (2). WSS angle was suggested to be an independent predictor for proximal aortic dilatation for patients with bicuspid aortic valves (33).

### Validation of WSS in CFD Point Clouds vs. Uniform Grid

Similar to other studies using deep learning, our training dataset was generated using CFD. While our target data is represented using a grid structure, we opted to train the network from the velocity sheets extracted directly from point clouds. Extracting velocity sheets from CFD point clouds allowed us to obtain smoother and velocity-rich information at flexible inward distances, unaffected by spatial sampling and any partial volume effect.

To showcase the generalizability of the network, we validated the network using a synthetic MRI dataset. The synthetic dataset was generated by sampling the CFD point clouds into MRI grid resolutions (dx = 2.4 and 1.2 mm). Additionally, we evaluated the robustness of the network in the presence of noise in the dataset. Subsequently, velocity sheets were extracted from the synthetic MRI, to simulate partial volume and discretization effects within the velocity sheets. Using WSSNet, reduced accuracy was identified at the velocity sheets acquired at a low resolution grid (dx = 2.4 mm). However, similar accuracy was observed with noise, showing the network is robust to noise. On the other hand, the parabolic fitting method shows much lower WSS values, and the values increase slightly with the presence of noise, which has been described previously (34). Increased accuracy was observed at higher spatial resolution (dx = 1.2 mm) for both methods. This is in agreement with previous studies that higher spatial resolutions lead to a higher average WSS (9–11). Nevertheless, WSSNet performance on low resolution synthetic MRI (dx = 2.4 mm) shows good accuracy and robustness to noise. Based on this validation on the synthetic MRI dataset, we can expect similar performance on the *in vivo* dataset with similar resolution.

To demonstrate the viability of our approach for 4D Flow MRI, we further tested the network in *in vivo* cases on base resolution (dx = 2.4 mm), similar to the test performed in the synthetic MRI. While there are no reference WSS values for *in vivo* cases, based on the previous validation on the synthetic MRI data (Figure 10), the regression and Bland-Altman plots in Figure 11 show similar trends. Moreover, visual observation and structural similarity (Pearson correlation) also show adequate results in terms of the WSS distribution.

In addition, Bland-Altman plots in Figures 10, 11 show similar trends with a previous study conducted by Cibis et al. (14), with ours showing a much larger bias. While their comparison shows the WSS difference between MRI and CFD resolution at the carotid arteries using the smoothing-spline fitting method, we show the difference between parabolic fitting and WSSNet at MRI resolution. This result demonstrates the network's capability to perform at a similar level of accuracy as CFD, with evaluation performed at MRI resolution only.

Compared with other factors (i.e., segmentation accuracy, venc), the spatial resolution had the most significant impact on WSS estimation, as shown by Petersson et al. (11), with WSS in MRI typically underestimating true WSS. In their study, the relationship between WSS estimation methods with voxel size, venc, and segmentation accuracy has been assessed extensively. Other non-velocity-based methods were also assessed but were outside the scope of this study.

Our average WSS in *in vivo* cases, using the parabolic fitting method (0.95 ± 0.46 Pa), are relatively similar to previous studies (1, 10). The differences are probably related to the different fitting methods (parabolic vs spline fitting). WSSNet shows a higher spatiotemporal average WSS of 2.95 ± 1.57 Pa. As we have shown in the synthetic MRI dataset, the accuracy of WSSNet is similar to CFD.

For the parabolic fitting method, despite the regression coefficients for TAWSS being low (at 2.4 and 1.2 mm), the correlations for OSI are much higher. It can be observed from Figure 10 that the systematic WSS underestimations (9) lead to a low correlation. However, OSI is a dimensionless metric measuring the changes in WSS direction, relative to its own magnitude. Therefore, it is independent of the WSS magnitude. As a result, the correlation of OSI between different methods can be compared independently from the magnitudes. This can be observed from the visualization of TAWSS and OSI (Figure 9) where both methods show TAWSS in different scales, but show OSI at the same scale and have a similar distribution.

Consistent regression coefficients for both methods compared to CFD reference values, in terms of TAWSS and OSI can be seen on the synthetic MRI dataset, with the higher resolution data showing a slight increase in correlation. The addition of noise to the data only affected the results slightly. Similar to the synthetic MRI, we also observed a similar correlation between the two methods for both TAWSS and OSI (Figure 11). This further verifies that WSSNet can be applied to *in vivo* cases, and exhibited similar behavior as when it was applied to synthetic MRI cases.

Additionally, Figures 10, 11 also show that the TAWSS estimates were higher in synthetic MRI (0–12 Pa) compared to the *in vivo* cases (0–8 Pa). These differences are likely caused by the choice of simplified boundary conditions (constant pressure at the outlet, outflow ratio) which produced different flow characteristics. However, despite the fact that the *in vivo* predictions are lower, the network can adapt to different flow patterns and still estimate WSS values accurately, since it is trained on a variety of patient-specific geometries and boundary conditions with the time-varying flow.

The choice of turbulence model also affects the CFD simulations and estimated WSS as ground truth data. Different turbulence models might produce different results, but the effect is likely to be small. The use of a laminar model is also an option, however, the WSS computed by a laminar model is known to be underestimated in the turbulent regime. Also, as observed in previous studies (35, 36), laminar models give rise to lower WSS estimates than turbulence models, which are less accurate for peak WSS estimates. Additionally, while the estimates might introduce differences in values, the WSS patterns are similar.

### Importance of WSS Distribution Pattern

Time-averaged WSS (TAWSS) and OSI patterns have been suggested as disease risk indicators, such as for atherosclerosis and aortic dilatation (1, 5, 37). Callaghan et al. (1) presented a normal pattern and range of WSS from 4D Flow MRI across a large population. Higher WSS was observed in the descending aorta compared with the aortic arch. In addition, this study suggested that WSS values are highly dependent on velocity, vessel diameter, and the aortic arch curvature. As previously mentioned, other studies have shown high accuracy in estimating hemodynamic variables, such as ECAP, based only on geometric information (spatial coordinates and curvature) (16, 17). While the presented results are accurate in *in silico* cases, pattern distribution and similarity were moderate. Liang et al. (18) also leveraged a geometric approach, with the predicted results showing similar aortic stress distributions, though it was not quantified and was only tested against finite element models. Another study combined vessel diameter and curvature information, showing good time-averaged WSS predictions in coronary arteries, with pattern similarities compared through visual inspection (15). Pattern matching is typically performed by checking areas of overlap using bins, categorizing WSS magnitudes from low to high (14).

In our study, we chose to use both velocity and geometric information, which is not only able to predict averaged or spatial results but can also predict spatiotemporal WSS. Moreover, our method is optimized using SSIM loss to ensure the network is able to recognize patterns based on spatial and velocity information. SSIM is a commonly used metric for image processing and computer vision, which can be applied to image data representation. Our results showed that WSSNet is able to recover finer WSS pattern details only by a two-fold resolution increase, which by no means is sufficient to accurately estimate WSS values. A much higher resolution is typically necessary to resolve the high gradient changes near the wall.

Overall, recent studies have suggested that WSS is a potential biomarker for atherosclerosis, aortic dilatation, and aneurysm (1, 5, 37). WSS measurement also has improved risk stratification in patients with carotid and coronary artery disease (38). Despite its clinical relevance, the available methods mostly rely on CFD analysis, which is computationally costly and not practical for a clinical setting (39). Therefore, by developing this method, we aim to extract the implicit knowledge from the CFD simulations, reducing a great deal of computation cost, while improving the applicability to a clinical setting. However, a more rigorous evaluation of patients is needed to ensure this approach can be applied for clinical applications.

### Limitations and Future Study

Our study has several limitations. First, a modest number of geometries were used to run the CFD simulations. Additional geometries could be used to improve the generalizability of the network. Furthermore, different boundary conditions may help to generate more variations in the training dataset.

Second, WSS computed using CFD are dependent on several assumptions, which may introduce errors. The choice of a turbulence model may also affect the calculation of WSS, due to how the CFD software approaches the calculation of wall viscosity (with the contribution of turbulence viscosity). Although there may be differences in WSS magnitudes introduced by different turbulent models, these differences are small and the WSS patterns are relatively similar. Furthermore, our approach relies on the dataset, where physical properties were inherently derived from the CFD simulations. The simplification of boundary conditions (e.g., plug profile, constant pressure) might have a significant impact on the patient-specific flow and WSS values (40). However, the impact on WSSNet results is likely to be small, as it learns from local velocities and spatial features.

Third, WSSNet requires a large amount of data to train. While it is possible to generate more data through CFD simulations, a more sustainable solution is needed. An alternative for CFD, such as Physics Informed Neural Network (PINN), enables physics-informed solutions to generate surrogate solutions faster than CFD (41). This approach may speed up the data generation process tremendously. Additionally, this method also allows direct estimation of WSS, which may solve this problem in one single step. However, this method requires retraining for each new geometry. In our case, where 4D Flow MRI datasets are already available, an algorithm like WSSNet offers a direct estimation of WSS using the available measurements and geometry information.

Finally, while representing the data as flatmaps saves computation power, and can be considered as a strength of this model, it is not a flexible representation. Using this representation, more complex geometries (including the aortic branches) cannot be represented as a rectangular grid using UV mapping. A more flexible data representation (i.e., mesh or graph) or a different network structure (i.e., SplineCNN, Graph Convolutional Networks) may open a lot more possibilities for more complex geometries (17, 42, 43). Graph data representations have the potential to remove completely the registration step from this workflow, which will improve the time and may be more readily applicable for a clinical setting.

To summarize, several future directions can be taken to extend the capability of this method, namely the addition of data to expand the network generalization capability, a more flexible data representation, and robustness to noise. Nevertheless, our study highlights the potential of combining geometric and velocity information in training deep neural networks to infer hemodynamic variables for 4D Flow MRI.

## Conclusion

In conclusion, we have presented a method to estimate WSS from 4D Flow MRI, with accuracy close to CFD. Our method is based on principles of similar previous WSS estimation methods, without being constrained by spatial resolution. More importantly, it is applicable to existing clinical MRI without any adjustments. We have shown accurate estimations for both CFD and *in vivo* cases regarding WSS magnitude and distribution throughout the aortic vessel.

## Data Availability Statement

The datasets generated for this study are available on request to the corresponding author.

## Ethics Statement

The studies involving human participants were reviewed and approved by Health and Disability Ethics Committees of New Zealand (HDEC 17/CEN/226). The patients/participants provided their written informed consent to participate in this study.

## Author Contributions

EF wrote the first draft, designed the network architecture, and performed the data preparation and analysis. CM contributed to the subdivision surfaces implementation. All authors participated in the conception and design of the study, interpretation of data, revision of the manuscript, and final approval of the submitted manuscript.

## Funding

This research has been funded by New Zealand Heart Foundation Scholarship, Grant No. 1786, the Health Research Council (HRC) of New Zealand, Programme Grants 17/608 and 21/144, as well as a grant from Siemens Healthineers, Erlangen, Germany.

## Conflict of Interest

Working expenses and a partial stipend for EF were provided by Siemens Healthineers, Erlangen, Germany. The remaining authors declare that the research was conducted in the absence of any commercial or financial relationships that could be construed as a potential conflict of interest.

## Publisher's Note

All claims expressed in this article are solely those of the authors and do not necessarily represent those of their affiliated organizations, or those of the publisher, the editors and the reviewers. Any product that may be evaluated in this article, or claim that may be made by its manufacturer, is not guaranteed or endorsed by the publisher.
